# Nine Months of Hybrid Intradialytic Exercise Training Improves Ejection Fraction and Cardiac Autonomic Nervous System Activity

**DOI:** 10.3390/sports11040079

**Published:** 2023-03-31

**Authors:** Christoforos D. Giannaki, Stefania S. Grigoriou, Keith George, Christina Karatzaferi, Paris Zigoulis, Eleftherios Lavdas, Dimitrios Chaniotis, Ioannis Stefanidis, Giorgos K. Sakkas

**Affiliations:** 1 Department of Life Sciences, University of Nicosia, Nicosia 2417, Cyprus; 2 Research Centre for Exercise and Nutrition (RECEN), Nicosia 2417, Cyprus; 3 School of Physical Education, Sport Science and Dietetics, University of Thessaly, 42100 Trikala, Greece; 4 Research Institute for Sport and Exercise Sciences, Liverpool John Moores University, Liverpool L2 2QP, UK; 5 Department of Medicine, School of Health Science, University of Thessaly, 38221 Larissa, Greece; 6 Department of Biomedical Sciences, University of West Attica, 12243 Athens, Greece; 7 School of Sports and Health Sciences, Cardiff Metropolitan University, Cardiff CF5 2YB, UK

**Keywords:** 2 d doppler echocardiography, exercise, cardiac function, hemodialysis

## Abstract

Cardiovascular disease is the most common cause of death in hemodialysis (HD) patients. Intradialytic aerobic exercise training has a beneficial effect on cardiovascular system function and reduces mortality in HD patients. However, the impact of other forms of exercise on the cardiovascular system, such as hybrid exercise, is not clear. Briefly, hybrid exercise combines aerobic and strength training in the same session. The present study examined whether hybrid intradialytic exercise has long-term benefits on left ventricular function and structure and the autonomous nervous system in HD patients. In this single-group design, efficacy-based intervention, twelve stable HD patients (10M/2F, 56 ± 19 years) participated in a nine-month-long hybrid intradialytic training program. Both echocardiographic assessments of left ventricular function and structure and heart rate variability (HRV) were assessed pre, during and after the end of the HD session at baseline and after the nine-month intervention. Ejection Fraction (EF), both assessed before and at the end of the HD session, appeared to be significantly improved after the intervention period compared to the baseline values (48.7 ± 11.1 vs. 58.8 ± 6.5, *p* = 0.046 and 50.0 ± 13.4 vs. 56.1 ± 3.4, *p* = 0.054 respectively). Regarding HRV assessment, hybrid exercise training increased LF and decreased HF (*p* < 0.05). Both conventional Doppler and tissue Doppler imaging indices of diastolic function did not change after the intervention period (*p* > 0.05). In conclusion, long-term intradialytic hybrid exercise training was an effective non-pharmacological approach to improving EF and the cardiac autonomous nervous system in HD patients. Such exercise training programs could be incorporated into HD units to improve the patients’ cardiovascular health.

## 1. Introduction

Cardiovascular disease is the leading cause of death in the hemodialysis (HD) population [1]. According to the literature, patients who receive HD therapy experience cardiac dysfunction and structure abnormalities [2], dysfunction of the autonomous nervous system and cardiac arrhythmias [1,3], as well as reduced cardiorespiratory fitness [4]. These factors are strongly associated with the high cardiovascular morbidity and mortality rate that characterizes the current HD population [1]. Previous research has suggested that the reduced heart rate variability (HRV) in HD patients may play an essential role in the higher risk of cardiovascular complications and sudden cardiac death [5].

Conventional HD therapy itself has been associated with various cardiovascular abnormalities and increased cardiovascular stress [1,6]. Intradialytic myocardial stunning (ischemia-mediated temporary reduction in cardiac function) may, over time, lead to irreversible fibrotic changes and chronic HF, arrhythmias, and sudden cardiac death (SCD) [7]. As HD is a frontline treatment option for end-stage renal disease and is required for these patients’ survival, interventions aiming to counterbalance its potential adverse effects on cardiovascular health are considered crucial.

It is well known that HD patients are usually physically inactive [8,9], and it seems that there is an association between low physical activity levels and all-cause mortality in this population [10]. Exercise training is considered to be an effective and safe non-pharmacological approach in terms of health, functional capacity and quality of life improvement in HD patients [11,12,13]. The most popular form of exercise for these patients is intradialytic aerobic exercise using cycle ergometers [13,14]. Aerobic exercise training has been shown to induce several beneficial effects on the cardiovascular system of HD patients, reducing cardiovascular events, improving autonomic function [15], increasing left ventricular ejection fraction [16,17], improving left ventricular mass [18], increasing cardiorespiratory fitness [13,19] and physical performance [20], improving stroke volume and cardiac output [17], reducing blood pressure [21] and improving their lipid profiles [22]. In addition, two recent studies showed that a single bout of intradialytic aerobic cycling reduced myocardial stunning [23,24].

Other forms of exercise, such as hybrid exercise, have been effective in improving overall health and quality of life parameters in patients with chronic diseases, including HD patients [25]. Briefly, a typical hybrid exercise session includes both aerobic (i.e., cycling) and resistance exercises (i.e., using elastic bands) and can also be implemented during the HD session [25]. A recent study from our group revealed that a single session with hybrid intradialytic exercise was well tolerated by HD patients and did not negatively affect left ventricular function during therapy [26]. The long-term effect of this form of exercise on cardiovascular risk profile, both at rest and during a HD session, has to be examined.

The present study examined whether hybrid intradialytic exercise has long-term benefits on left ventricular function and structure and autonomous nervous system in HD patients. All measures of HRV as well as LV structure and function were assessed at rest as well as during and after an acute HD therapy session, before and after the intervention period in patients on HD. The Echocardiographic scans were collected before the initialization of the HD session, during the last hour of the HD session and after the end of the HD session. The HRV parameters were collected prior to HD therapy, every one hour of HD therapy and after the end of the HD session. All parameters were collected while patients were resting on the bed.

## 2. Materials and Methods

### 2.1. Participants

**Figure 1:**
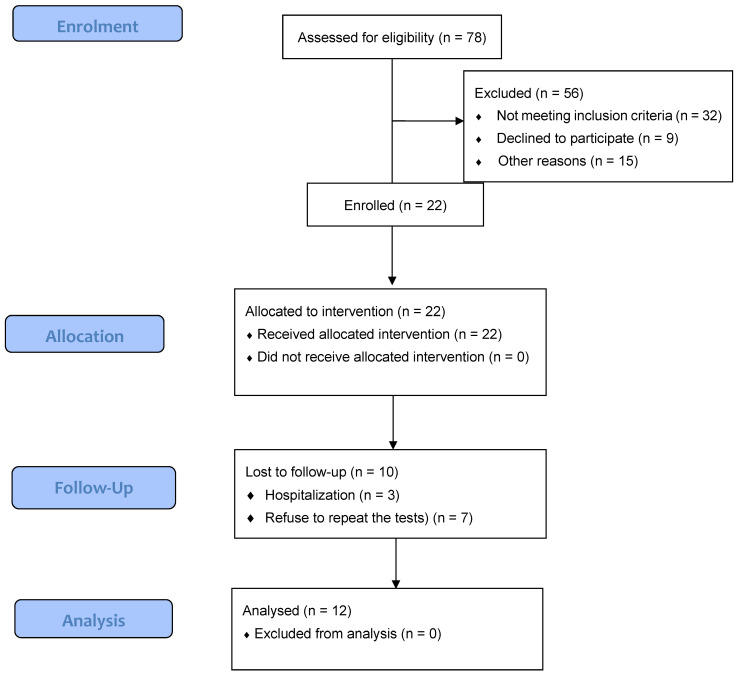
Flow of participants through the study.

Patients were recruited from the HD unit of the local hospital. The inclusion criteria for the study were: being on HD for at least three months or more with adequate dialysis delivery and with a stable clinical condition. The exclusion criteria included: (1) presence of diagnosed neuropathies (2) presence of a catabolic state within three months before the start of the study, (3) or unable or did not agree to participate in an exercise training program. None of the recruited patients were engaged in any systematic exercise training program 3 months prior to the initialization of the study. After the initial screening, twelve patients (10M/2F, 56 ± 19 years) fulfilled the criteria and enrolled in the study (Figure 1). The Human Research and Ethics Committee approved the study of the University of Thessaly, and it was approved by the bioethics committee of the University General Hospital of Larissa, Greece (UHL). All patients gave their written informed consent before study initialization. The whole study is registered at ClinicalTrials.gov (NCT01721551) as a clinical trial, while this current study presents a subset of data acquired under the registered RCT study.

### 2.2. Hybrid Intradialytic Exercise Program

Patients followed a 9-month intradialytic exercise training program supervised by two specialized exercise physiologists. With regards to the aerobic exercise program, supine cycle exercise was performed three times weekly for 60 min each time during the first 2 h of HD sessions using an adapted bicycle ergometer (Model 881 Monark Rehab Trainer, Varberg, Sweden) at an intensity of 50–60% of the patient’s maximal exercise capacity (in Watts), which was estimated during a previous HD session using a modified version of Åstrand Bicycle Ergometer test [27]. This test required the patient to cycle in the supine position at 50 rpm while the intensity was increased by 10 watts every 1 min until exhaustion. Afterward, the patients performed 20 min of resistance exercise using resistance bands (TheraBand© professional Latex, AKRON, OH 44,310, USA—Resistance from Green to Silver) and portable ankle weights and dumbbells. Briefly, the resistance training program consisted of 3 sets of 12 repetitions of the following exercises: (i) resistance bands exercises: chest press, triceps extension, shoulder flexion, hip abductions, seated row; (ii) ankle weights and dumbells exercises: knee extension, biceps curl, hip fexion, shoulder press, side shoulder raise, straight-legged raise. The hand with the fistula was excluded from any exercise. The resistance training intensity was assessed by the Borg Rating of Perceived Exertion (RPE) scale and set to be between 14 and 16 (medium to hard). The aerobic part was implemented at the beginning of the training bout followed by the resistance component. The interval between the two types was 10 min. The work-to-rest ratio regarding the resistance training was 1:1. Resting between sets and exercises included lying down doing nothing for 2 min. The exercise intensity and the resistance of the hybrid exercise program were assessed every 6 weeks and adjusted accordingly. In particular, the intensity of the aerobic part of the program was adjusted (in watts) based on the performance of the patients in the modified version of the Åstrand Bicycle Ergometer test. As mentioned in the text, the resistance training intensity was assessed by the Borg RPE scale and set to be between 14 and 16. When the patient reported values lower than 14, the resistance was re-adjusted by changing the type of the elastic bands (i.e., from Green to Blue, etc.) and increasing the dumbbell weight.

### 2.3. Hemodialysis Procedure

The patients underwent HD therapy (4 h × 3 times per week) (Fresenius 4008B, Oberursel, Germany) with low flux, hollow-fiber dialysers and bicarbonate buffers. An enoxaparin dose of 40–60 mg was administered intravenously before the beginning of each HD session. In addition, Erythropoietin therapy was given after the completion of the HD session in order to normalize hemoglobin levels within 11–12 (g/dL).

### 2.4. Echocardiography

Echocardiographic scans were performed by an experienced cardiologist-echocardiographer using an iE33 echocardiographic system (Philips Medical Systems, Andover, MA, USA). All image acquisitions were made with the subject lying in the left lateral decubitus position using a 2.5 MHz transducer. Three consecutive beats were analyzed in each scan for each patient, and the mean value was used in the subsequent statistical analysis. A single-lead ECG inherent to the echocardiographic system was used for the recording of HR. Left ventricular (LV) dimensions were determined from 2-dimensional guided M-Mode images according to the American Society of Echocardiography (ASE) recommendations for chamber quantification [28] using the parasternal long-axis acoustic window. LV mass was calculated from M-Mode traces at the mitral valve level and determined in g by using the recommended ASE formula. LV mass index was calculated by dividing LV mass by body surface area (using the DuBois and DuBois formula) and height to minimize the effects of age, gender, and overweight status [28]. For the assessment of LV diastolic function, the transducer was applied apically (4-chamber view) whilst a pulsed wave Doppler sample volume (4 mm) was located at the tips of the mitral valve leaflets. Doppler gain, pulse repetition frequency, and high-pass filter were all adjusted to maximize the signal-to-noise ratio. The following parameters were evaluated: early peak flow velocity (E), late peak flow velocity (A); thus, the ratio of E to A was calculated. The ejection fraction was calculated using the biplane Simpson’s method from 2-dimensional apical 2- and 4- chamber orientation to evaluate the patient’s systolic function. Tissue Doppler velocities were assessed at the basal septum, using pulsed-wave Doppler. The sample volume (2 mm) was placed at the basal septum at the level of the mitral annulus ring in parallel to the longitudinal movement of the septum. Peak early diastolic (E’) and peak late diastolic (A’) myocardial tissue velocities were assessed and the E’/A’ ratio was calculated. In addition, the conventional Doppler E to tissue Doppler E’ ratio (E/E’) was calculated.

### 2.5. Heart Rate Variability Assessment

Heart rate variability was measured using heart rate monitors (RS800CX, Polar Electro Oy, Kempele, Finland) validated for heart rate variability assessment [29]. For the heart rate variability time domain, the square root of the mean of squared differences between successive RR intervals and the percentage of successive normal-to-normal intervals greater than 50 milliseconds were computed [30]. For the HRV frequency domain, the low and high-frequency bands, expressed in normalised units (nu) and their ratio (low frequency/high frequency) were reported [30]. HRV indices (low-frequency activity, high-frequency activity, low-frequency/high-frequency activity, the square root of the mean of squared differences between successive RR intervals and the percentage of successive normal-to-normal intervals greater than 50 milliseconds) were analyzed using Kubios Heart Rate Variability Analysis Software V1.1 (Kubios Oy, Business ID 2740217-3,Varsitie 22, 70150 Kuopio, FINLAND). The HRV parameters were collected prior to HD therapy, every one hour of HD therapy and after the end of the HD session.

### 2.6. Blood Chemistry

Routine monthly biochemical results were recorded, including C reactive protein, ferritin, iron, hematocrit, and hemoglobin. The analyses were performed at the clinical biochemistry lab of the University Hospital of Larissa under standard hospital procedures.

### 2.7. Statistical Analysis

Statistical analysis was performed using one-way repeated-measures analysis of variance (ANOVA). When ANOVA showed statistical significant differences between measurements, Bonferroni’s correction for multiple comparisons was performed to assess where specific differences occurred. In addition, for comparing initial and final values (pre and post-exercise training), paired t-tests were used. The results are expressed as mean ± SD. All the statistical analysis was performed using the Statistical Package for the Social Sciences (SPSS for Windows, version 18.0, Chicago III). The level for statistical significance was set at *p* ≤ 0.05.

## 3. Results

All twelve HD patients completed the 9-month intervention program without any adverse effects. Patient basic characteristics are presented in Table 1.

Echocardiographic data are presented in Table 2. Significant improvements were observed in the EF after intradialytic exercise both at baseline and after the nine-month intervention period, whilst the pre-HD value of EF appears to be significantly improved when assessed at baseline after the nine months of exercise training compared to the baseline value (*p* = 0.046). Both conventional Doppler and TDI indices of diastolic function did not change after the intervention period (*p* > 0.05). Finally, regarding the measurement performed at the end of the HD session, EF% (*p* = 0.054) and DT (*p* = 0.014) increased and decreased, respectively, after the intervention.

HRV indices are presented in Table 3. LF reduced and HF increased, while pNN50% was higher after the intervention (*p* < 0.05).

## 4. Discussion

This study investigated the effect of a 9-month hybrid exercise training regimen, undertaken during HD sessions, on left-ventricular function and structure and HRV (both assessed at rest, during the HD therapy and after the end of the HD session) in hemodialysis patients. The findings of our study demonstrated that long-term hybrid intradialytic exercise did not negatively impact the ejection fraction or HRV, and possibly could improve left ventricular function and HRV. These outcomes bear high clinical significance as HD patients are vulnerable to cardiovascular problems and increased mortality.

The present study showed that nine months of hybrid exercise during HD sessions significantly increased resting LV EF. Possible mechanisms explaining the increase of EF after systematic exercise training include increased oxygen supply of cardiac muscle, reduction in cardiac afterload and augmented function of cardiac autonomic nervous system activity [17,22,31]. The current study’s findings of positive changes in EF with a hybrid exercise intervention confirm and extend previous studies using long-term aerobic intradialytic exercise training [17]. For instance, in the study by Deligiannis and colleagues, six months of intradialytic aerobic exercise resulted to a 5% increase in resting EF [17]. An approximately 4% increase in resting EF was observed in the present study. Therefore, similarly to pure aerobic exercise, hybrid intradialytic exercise could have cardioprotective properties (as it uses similar aerobic exercise exposure in the aerobic part of the exercise regimen) and thus could be recommended to these patients.

There is some evidence revealing beneficial effects of intradialytic exercise on HD therapy itself such as improved HD efficiency and increased solute removal [20,32], reduced motor restlessness [33] as well as improved psychological parameters [34], post-dialysis fatigue [25] and sleep quality [35]. Similarly, with acute intradialytic hybrid exercise [26], the application of nine months of the same exercise form did not result in improvements or impairments in left ventricular diastolic function parameters when assessed during the HD therapy. Future studies may explore the effect of even longer and different (i.e., using higher intensities) interventions using hybrid exercise on cardiovascular parameters during the HD therapy.

In the present study, the HF and the LF parameters were found to be significantly increased and decreased, respectively, after nine months of exercise training; they showed a favorable adaptation to exercise of cardiac autonomic nervous system activity. According to the literature, exercise training can reduce emotional distress and concomitantly improve HRV [36], reducing susceptibility to arrhythmias [37]. Our findings bear a high clinical significance as in previous studies; a reduction in the SDNN, LF, and LF/HF parameters that predicted cardiovascular death and, more specifically, sudden death [38].

A large body of evidence shows that HD patients are vulnerable to cardiovascular diseases and have very high mortality. Although many studies reveal that exercise can improve the functionality of various physiological systems and overall health, most HD patients are physically inactive [8] and they do not participate in exercise training programs, despite having a positive perception of exercise [39]. Hybrid exercise is a relatively new form of training that combines aerobic with resistance exercise. The current study’s findings support intradialytic exercise training programs as non-pharmacological methods to improve cardiovascular system functionality in HD patients, introducing this form of exercise as an alternative to traditional aerobic exercise. Hemodialysis units can be ideal settings for delivering safe and effective exercise programs for the patients, improving health and quality of life parameters, while the incorporation of exercise professionals into these units could help the patients to engage in exercise interventions.

We must acknowledge that some studies did not report significant improvements in HRV and left ventricular function after aerobic intradialytic exercise training [40]. This can be attributed to both the reduced duration of the intervention compared to the current study and differences in the nature of exercise training (aerobic vs hybrid). The current study has some strengths and weaknesses that we wish to acknowledge. The main limitation of the study is the lack of a control group. This was a single-group design, efficacy-based nine-month intervention, and the participants were recruited from a single HD unit, thus it was difficult to find patients who were willing to undergo the examination without doing exercise. Patients on hemodialysis are unique and require continuous and extensive care to keep active and healthy, so such long-term interventions are not common. On the other hand, the study’s strengths were the long duration of the supervised exercise program and the echocardiographic examination that was performed, among other points, during the HD session (a very challenging and demanding procedure). Randomized-controlled trials, with larger sample sizes, need to be conducted in the future to compare the effectiveness of hybrid exercise over other traditional forms of exercise on this specific population.

In conclusion, nine months of supervised hybrid intradialytic exercise training did not negatively impact the ejection fraction or heart rate variability indices. On the contrary, it seems that the combination of aerobic and resistance training in a single bout of exercise has a positive effect on ejection function and heart rate variability in stable hemodialysis patients. Hybrid intradialytic exercise training is well tolerated and could be suggested as a non-pharmacological approach for improving cardiovascular health in hemodialysis patients.

## Figures and Tables

**Table 1:** Patients basic characteristics before and after nine months of intradialytic exercise training.

Variables	Pre	Post 9 Months
N	12	12
Female/Male	2/10	
Age (year)	56 ± 19	57 ± 17
Dry Weight (kg)	73.2 ± 16.4	75.4 ± 16.9
Height (m)	1.69 ± 0.10	1.69 ± 0.10
BMI (kg/m^2^)	26.1 ± 5.2	26.9 ± 5.5
Months in dialysis	40 ± 44	
WHR	1.02 ± 0.12	1.00 ± 0.1
CRP (mg/dL)	3.7 ± 5.6	0.8 ± 0.5
HCT	34.7 ± 4.0	34.2 ± 3.2
Hb(g/dL)	11.2 ± 1.3	10.8 ± 1.0
Iron(μg/dL)	58.2 ± 30.8	51.7 ± 27.2
Ferritin (ng/mL)	1377.3 ± 1170.4	754.3 ± 518.7

All data are mean ± SD. BMI, Body mass index; WHR, waist-to-hip ratio; CRP, C Reactive Protein; HCT, hematocrit; Hb, hemoglobin.

**Table 2:** Echocardiographic indices pre and post the exercise training intervention.

Parameter	Scenario	Pre HD	During HD	Post HD
Standard Echocardiographic Indices				
IVSTd(mm)	Pre	* 11.9 ± 2.2	11.1 ± 2.3	10.4 ± 1.8
	Post 9 months	* 9.9 ± 2.3	11.0 ± 4.2	9.9 ± 2.5
Cohens’s d		0.88	0.02	0.22
LVPWTd (mm)	Pre	11.0 ± 2.4	10.4 ± 2.4	9.9 ± 1.8
	Post 9 months	9.9 ± 2.4	9.3 ± 2.5	9.8 ± 2.1
Cohens’s d		0.45	0.44	0.05
LVIDd(mm)	Pre	45.5 ± 4.6	28.8 ± 4.0	44.8 ± 4.5
	Post 9 months	48.0 ± 6.2	45.6 ± 5.7	46.9 ± 5.6
Cohens’s d		−0.45	−3.41	−0.41
LV mass(g)	Pre	57.8 ± 9.0	54.2 ± 10.7	51.7 ± 6.9
	Post 9 months	55.4 ± 10.2	56.5 ± 15.9	55.1 ± 10.2
Cohens’s d		0.24	−0.16	−0.39
LV mass/BSA(g/m^2^)	Pre	31.7 ± 3.8	26.7 ± 10.0	28.2 ± 4.3
	Post 9 months	29.7 ± 4.4	30.0 ± 5.8	29.3 ± 5.5
Cohens’s d		0.48	−0.40	−0.22
LV mass/height^27^	Pre	14.7 ± 2.2	12.3 ± 4.8	12.9 ± 2.1
	Post 9 months	14.1 ± 2.9	14.0 ± 3.7	13.6 ± 3.1
Cohens’s d		0.23	−0.39	−0.26
EF (%)	Pre	* 48.7 ± 11.1	52.8 ± 10.1	* 50.0 ± 13.4
	Post 9 months	* 58.8 ± 6.5	60.4 ± 10.1	* 56.1 ± 3.4
Cohens’s d		−1.11	−0.75	−0.61
Doppler Mitral Inflow Indices				
E (mm/s)	Pre	0.8 ± 0.2	0.6 ± 0.13	0.7 ± 0.1
	Post 9 months	0.8 ± 0.2	0.6 ± 0.2	0.7 ± 0.2
Cohens’s d		0	0	0
A (mm/s)	Pre	0.9 ± 0.2	0.8 ± 0.3	0.9 ± 0.3
	Post 9 months	0.9 ± 0.3	0.8 ± 0 3	0.8 ± 0.3
Cohens’s d		0	0	0.33
E/A	Pre	0.9 ± 0.2	0.9 ± 0.3	1.0 ± 0.4
	Post 9 months	1.1 ± 0.4	0.9 ± 0.4	0.9 ± 0.3
Cohens’s d		−0.63	0	0.28
DT (ms)	Pre	** 250.9 ± 48.0	255.3 ± 70.2	** 261.1 ± 61.9
	Post 9 months	** 192.3 ± 41.4	228.5 ± 43.1	** 215.0 ± 50.5
Cohens’s d		1.30	0.46	0.81
IVRT(ms)	Pre	62.1 ± 12.4	56.6 ± 12.6	63.8 ± 13.2
	Post 9 months	73.4 ± 13.3	69.4 ± 17.8	71.6 ± 14.7
Cohens’s d		−0.87	−0.83	−0.55
Tissue Doppler Myocardial Velocities Indices				
E’ (mm/s)	Pre	0.1 ± 0.0	0.1 ± 0.0	0.1 ± 0.0
	Post 9 months	0.1 ± 0.0	0.1 ± 0.0	0.1 ± 0.0
Cohens’s d		0	0	0
A’ (mm/s)	Pre	0.9 ± 0.0	0.1 ± 0.0	0.1 ± 0.0
	Post 9 months	0.1 ± 0.0	0.1 ± 0.0	0.1 ± 0.1
Cohens’s d		0	0	0
E’/A’	Pre	1.0 ± 0.4	0.9 ± 0.5	0.8 ± 0.4
	Post 9 months	1.3 ± 1.1	0.8 ± 0.3	1.1 ± 0.7
Cohens’s d		−0.36	0.24	−0.52
E/E’	Pre	10.2 ± 3.5	8.1 ± 2.7	10.0 ± 4.1
	Post 9 months	8.4 ± 3.4	8.6 ± 3.7	7.5 ± 3.7
Cohens’s d		0.52	−0.15	0.64

All data are mean ± SD. HD, hemodialysis; IVSTd, interventricular septum thickness in diastole; LVPWTd, left ventricular posterior wall thickness in diastole; LVIDd, left ventricular internal diameter in diastole; LV, left ventricle; BSA, body surface area; EF, ejection fraction; E, early diastolic mitral flow velocity; A, late diastolic mitral flow velocity; E/A, ratio of early to late diastolic flow velocity; DT, deceleration time; IVRT, isovolumic relaxation time, E’, early mitral annular velocity, A’, late mitral annular velocity; E’/A’, ratio of early to late mitral annular velocity; E/E’, ratio of early mitral flow velocity to early mitral annular velocity. ** significant differences at the 0.01 level (2-tailed). * significant differences at the 0.05 level (2-tailed).

**Table 3:** Heart Rate Variability indices before and after nine months of intradialytic exercise training.

		Pre HD	1	2	3	4	End of HD
SDNN(ms)	Pre Post 9 months	64.03 ± 44.1 48.4 ± 19.8	74.5 ± 32.2 55.7 ± 22.9	58.8 ± 32.9 62.9 ± 35.2	55.1 ± 35.1 63.8 ± 38.4	53.8 ± 30.9 96.7 ± 79.6	52.5 ± 31.5 164.8 ± 241.0
Cohens’s d		0.45	0.70	−0.12	−0.23	−0.71	−0.65
mean RR interval (ms)	Pre Post 9 months	* 838.6 ± 93.3 802.7 ± 70.8	800.1 ± 103.6 805.9 ± 79.6	763.2 ± 121.4 811.3 ± 96.9	711.0 ± 154.5 807.4 ± 88.0	730.4 ± 156.1 805.3 ± 99.3	718.7 ± 155.6 799.8 ± 103.7
Cohens’s d		0.43	−0.06	−0.43	−0.76	−0.57	−0.61
LF (ms^2^)	Pre Post 9 months	^#^ 67.2 ± 16.5 58.9 ± 20.1	68.0 ± 12.9 67.8 ± 17.5	69.2 ± 10.8 67.2 ± 70.7	68.4 ± 18.6 67.2 ± 16.4	67.6 ± 22.8 62.2 ± 17.3	71.3 ± 22.6 66.1 ± 25.2
Cohens’s d		0.45	0.01	0.03	0.06	0.26	0.21
HF (ms^2^)	Pre Post 9 months	^#^ 32.8 ± 16.5 41.1 ± 20.1	32.1 ± 12.9 32.2 ± 17.5	30.8 ± 10.8 32.8 ± 16.4	31.6 ± 18.6 32.8 ± 16.4	32.4 ± 22.8 37.8 ± 17.3	28.7 ± 22.6 33.9 ± 25.2
Cohens’s d		−0.45	−0.00	−0.14	−0.06	−0.26	−0.21
LF/HF ratio	Pre Post 9 months	2.7 ± 1.5 2.1 ± 1.7	2.7 ± 1.5 2.9 ± 1.8	2.7 ± 1.5 2.9 ± 2.4	3.5 ± 2.7 5.3 ± 7.0	3.6 ± 2.6 2.2 ± 1.3	5.2 ± 5.2 8.2 ± 17.6
Cohens’s d		0.37	−0.12	−0.09	−0.33	0.68	−0.23
rMSSD(ms)	Pre Post 9 months	42.7 ± 67.2 31.1 ± 17.2	32.0 ± 23.3 42.6 ± 34.3	24.0 ± 12.1 52.3 ± 56.9	20.5 ± 17.5 49.7 ± 58.8	31.8 ± 37.3 76.7 ± 102.2	28.8 ± 31.1 79.8 ± 79.7
Cohens’s d		0.23	−0.36	−0.68	−0.67	−0.58	−0.84
pNN50%	Pre Post 9 months	7.7 ± 11.4 10.3 ± 13.2	^#^ 5.8 ± 4.3 6.3 ± 7.4	4.5 ± 3.9 10.0 ± 15.1	6.6 ± 12.3 10.0 ± 15.1	9.1 ± 14.8 10.3 ± 15.8	8.1 ± 12.1 9.5 ± 13.7
Cohens’s d		−0.21	−0.08	−0.49	−0.24	−0.07	−0.10

All data are mean ± SD.SDNN; standard deviation of the normal RR intervals, mean RR interval; mean duration of all normal to normal RR intervals, LF; low-frequency component, HF; high-frequency component, rMSSD; square root of mean squared forward differences of successive NN intervals, pNNS0; proportion of successive NN intervals differences > 50. * Differences between hours; ^#^ Differences between pre and post-exercise intervention.

## Data Availability

All data and materials of this study are available upon request.
